# A Review of the Relationship Between Social Media Use and Online Prosocial Behavior Among Adolescents

**DOI:** 10.3389/fpsyg.2021.579347

**Published:** 2021-09-28

**Authors:** Christoffer Lysenstøen, Tormod Bøe, Gunnhild Johnsen Hjetland, Jens Christoffer Skogen

**Affiliations:** ^1^Department of Psychosocial Science, Faculty of Psychology, University of Bergen, Bergen, Norway; ^2^Regional Centre for Child and Youth Mental Health and Child Welfare, NORCE Norwegian Research Centre, Bergen, Norway; ^3^Department of Clinical Psychology, Faculty of Psychology, University of Bergen, Bergen, Norway; ^4^Department of Health Promotion, Norwegian Institute of Public Health, Bergen, Norway; ^5^Alcohol & Drug Research Western Norway, Stavanger University Hospital, Stavanger, Norway; ^6^Department of Public Health, Faculty of Health Sciences, University of Stavanger, Stavanger, Norway

**Keywords:** online prosocial behavior, social media, systematic review, Newcastle-Ottawa scale, adolescents

## Abstract

Social media (SoMe) activity constitutes a large part of the lives of adolescents. Even though the behavior on SoMe is complex, the research on SoMe has mostly focused on negative effects, bad content, and online antisocial behavior (OAB). Less research has been conducted on online prosocial behavior (OPB), and to what extent OPBs are widespread is relatively unknown. A review was conducted to investigate to what extent OPB is related to SoMe use among adolescents based on studies published from 2014 to May 2021. To be included, the studies had to be quantitative, non-experimental, have participants aged 13–18, include measures of SoMe and OPB, and be published in peer-reviewed journals with full text available in English, Swedish, Danish or Norwegian. A research was conducted in databases PsychINFO, Ovid MEDLINE(R), EMBASE, COCHRANE Database of Systematic Reviews, Web of Science, Sociological Abstracts, Sociological Services Abstracts, and Eric. Two studies met the eligibility criteria. Both studies found an association between OPB and SoMe use. Methodological issues, however, were identified through a quality assessment using an adapted version of the Newcastle-Ottawa Scale (NOS) for cross-sectional studies, and the small samples in the studies prevent us from drawing any firm conclusions. Possible reasons for the scarcity of eligible studies and directions for future research are discussed.

**Systematic Review Registration:** PROSPERO; ID CRD42020162161 and CRISTIN; ID 2038994.

## Introduction

Social media (SoMe) have been defined as websites, services, and related tools that allow participants to create and share their content (Boyd, 2014). An estimated 3.48 billion people were using SoMe worldwide in 2019 and an increase of 9% since 2018 (Kemp, 2019).

Adolescents are among the most active users, and the 2018 Pew Report showed that almost half of all U.S. teenagers report being online “almost constantly,” and 87% report using at least one SoMe platform daily (Pew Research Center, 2018). Social networking sites dominate the landscape, with Facebook, Twitter, and Instagram being the most popular sites. Instant messaging services (e.g., Snapchat and WhatsApp) have recently overtaken a substantial part of the userbase, with reports showing over one-third of adolescents using Snapchat more often than the larger social networking sites (Pew Research Center, 2018). Lastly, vlogging sites, sites where adolescents can upload or stream personal content for others to react, share, and respond to (i.e., YouTube), are also widely popular among youth (Pew Research Center, 2018).

A growing concern has been raised by several researchers regarding the potential negative effects of SoMe use (Han, 2018; Twenge and Campbell, 2019). SoMe use has especially been linked to mental health problems, and one meta-analysis found an association between social networking use and depression and anxiety (Keles et al., 2020). Others have found both negative and positive associations with well-being (Verduyn et al., 2017).

Much of the previous research on SoMe has focused on its possible *effects* (Orben, 2020; Schønning et al., 2020), while some studies focus on drivers for SoMe use and screen-based activities (Scott et al., 2019; Thomas et al., 2020), such as fear of missing out. Another area of study has been the *type of behavior* children and adolescents perform on SoMe (Kircaburun et al., 2019). The type of online social behavior, as opposed to more general measures, such as “time-spent on SoMe” or “amount of screen time-activity,” might influence the associations with outcome variables (i.e., mental health and well-being). Indeed, a great deal of attention has been directed at the negative behavior performed online by adolescents, typically in the form of cyberbullying (Kowalski et al., 2014). Cyberbullying is quite common (Brochado et al., 2016) and can have a serious impact on children and adolescents, as it is linked to depression, anxiety, lower self-esteem, and academic performance both for the bullies and the bullied (Kowalski and Limber, 2013).

A recent scoping review on SoMe use and mental health and well-being among adolescents concluded that most previous studies have focused on negative aspects of SoMe use (Schønning et al., 2020). Less research seems to have been devoted to positive aspects of SoMe (Schønning et al., 2020), such as online prosocial behavior (OPB). To our knowledge, there are no reviews on OPB; there is only one comprehensive book chapter by Wright and Li (2012). For comparison, a systematic map of reviews on screen-based activities and mental health outcomes of children and adolescents found 19 reviews on cyberbullying, whereby included primary studies in each review ranged from 10 to 131 (Dickson et al., 2018). Within research on SoMe use, it can be argued that cyberbullying is so widely researched that it constitutes its own research domain (Schønning et al., 2020). Thus, the potential aspects of SoMe use (Schønning et al., 2020) and OPB seem to be under-researched, and little is known about OPB of adolescents today.

However, a wealth of research has been conducted on offline (i.e., traditional) prosocial behavior since the 1970s (Eisenberg et al., 2007). Prosocial behavior has conventionally been defined as voluntary actions intended to benefit others (Eisenberg et al., 2007). Such behaviors can be helping, comforting, sharing with, and supporting others. Prosocial behaviors can be motivated by a variety of factors, such as getting a reward, gain approval from others, acting according to social norms, or out of genuine sympathy (Eisenberg and Mussen, 1989). Studies have found that offline prosocial behavior is associated with several positive outcomes, such as better academic performance (Carlo et al., 2018), higher self-esteem (Laible et al., 2004), and subjective well-being (Aknin et al., 2013). Experimental research shows that performing prosocial behaviors can lead to feelings of well-being and happiness (Aknin et al., 2013; Martela and Ryan, 2016). These findings warrant a greater interest in the online counterpart of prosocial behavior.

### Online Prosocial Behavior

Online prosocial behavior, or cyberprosocial behavior (Wright and Li, 2012), refers to prosocial behavior in a digital context (i.e., while being on the internet). As previously mentioned, only one book chapter (Wright and Li, 2012) has attempted a summary of the research on OPB, and no reviews exist. We argue that the need for an updated review is warranted for several reasons.

First, the chapter by Wright and Li (2012) compiled much of the seemingly relevant research on OPB, yielding a wide picture, unable to draw practical conclusions or future directions. The chapter details a historical account of OPB, starting with prosocial behavior during the pre-internet bulletin board systems, in the 1980s (Schneider, 1986), up to prosocial behaviors on social networking sites (Wright and Li, 2011). The authors operated with a wide definition of prosocial behaviors, such as online mentoring, donating to online charities, virtual voluntarism, helping through electronic groups, social networking services, and online gaming. Such a wide definition of prosocial behaviors on SoMe today may be too wide as it may encapsulate inherently different forms of prosocial behaviors. Evidence suggests that there are different forms of helping and that they may differ on the basis of motivation, targets, and outcomes (Carlo and Randall, 2002; Carlo et al., 2003; Padilla-Walker and Carlo, 2015). For instance, motivations behind prosocial behavior may be altruistic or egoistic. While altruistic prosocial behavior would for instance entail helping someone despite personal costs, egoistic prosocial behavior would mean doing certain good deeds to get a good conscience. Thus, OPBs directed at individuals compared to prosocial behaviors directed at organizations and large groups (e.g., donating or voluntarism) may differ substantially. One can for example argue that donations and voluntarism are closely linked to civic engagement and political orientation in general and not OPB *per se*. In addition, voluntary organizations and political parties commonly invest in commercials and other activities aimed at soliciting specific (prosocial) behaviors from potential contributors, such as donations. To obtain a more specified account of OPB of the adolescents, this review seeks to investigate OPB directed at particular others, excluding donations and voluntarism, and including forms of communication between individuals online.

Second, although the chapter by Wright and Li (2012) was comprehensive, the studies enlisted may no longer be generalized or relevant, due to the continuous and enormous evolution of SoMe during the last 15 years. As the review detailed studies conducted in the interval from 1980 to 2011, with the majority of them being conducted prior to 2005, many of the studies missed the advent of Facebook in 2004 (Facebook, 2020) and smartphones, particularly the iPhone in 2007^1^. Arguably, the landscape of SoMe and the size of its userbase have transformed since 2005. Thus, there is a clear need for a new and updated review.

Third, OPB is arguably in need of research attention, as the research on offline prosocial behavior yield findings contributing to adolescent well-being and happiness. In their book chapter, Wright and Li (2012) outline that cyberprosocial behavior may result in the same benefits as offline prosocial behavior, both for the receiver (Brennan et al., 1992; Sudzina et al., 2015) and for the helper (Mukherjee, 2010; McAleer and Bangert, 2011), indicating the need for more research on the topic.

Fourth, just as the potential for harmful behaviors on the internet is ample (i.e., cyberbullying), the potential for prosocial behaviors is also extensive. Content analyses of online messages in blogs, chats, and social networks indicate the ominous presence of prosocial behaviors in terms of empathic and supportive comments and messages (Baym, 2002; Thelwall et al., 2010). The cyber context contains an abundance of possible helpers and receivers, and a variety of prosocial behaviors are being performed and received on SoMe. Adolescents use SoMe to give and receive support from informal peer networks (Gibson and Trnka, 2020), but also from strangers (Gibson, 2016), to share emotions and to respond aptly to emotion sharing (Bazarova et al., 2015; Vermeulen et al., 2018), to help each other when playing online games (Wang and Wang, 2008), and cooperate with adolescents they identify with (Kim and Kim, 2017). They are more willing to confide in friends than in adults and professionals (Michelmore and Hindley, 2012), indicating that a lot of OPBs remain unnoticed by parents, teachers, and other authority figures in their lives. Most of this research is qualitative, using focus groups or interviews, with a low number of respondents. Thus, it is hard to form a comprehensive overview of to what degree the time of adolescent on SoMe concerns OPB.

To form a more concrete and comprehensive overview, this paper aimed to conduct a systematic review on the relationship between SoMe use and OPB among adolescents.

#### Definitions

We used the following definition offered by Kietzmann et al. (2011, p. 1): “Social media employ mobile and web-based technologies to create highly interactive platforms via which individuals and communities share, co-create, discuss, and modify user-generated content” (see Kietzmann et al., 2011, for a comprehensive account).

Online prosocial behavior refers to “voluntary behavior carried out in an electronic context (/social media context) with the intention of benefitting particular others or promoting harmonious relations with others” (Erreygers et al., 2018a). Examples of OPB include comforting a friend via digital technologies, online sharing of resources and information with a classmate, and helping peers out on social network sites. This definition excludes behaviors, such as online donations to charities, online volunteering, and helping online organizations, as the definition of OPB focuses on *particular others* and thus the relational nature of adolescent behavior (Erreygers et al., 2018a).

## Methods

### Protocol and Registration

The protocol for this review was registered with the International Prospective Register of Systematic Reviews on December 12, 2019 (PROSPERO; ID CRD42020162161). It has also been registered with the Current Research Information System in Norway (CRISTIN; ID 2038994). This paper follows the PRISMA guidelines and uses a systematic approach to the review process (see Appendix A).

### Search Strategy and Databases

The databases PsychINFO, Ovid MEDLINE(R), EMBASE, COCHRANE Database of Systematic Reviews, Web of Science, Sociological Abstracts and Sociological Services Abstracts, and Eric were systematically searched on December 9 and 10, 2019. For an example of search strategy, see Table 1 (the complete search strategy for all databases are available in Appendix B).

**Table 1:** Example of search strategy.

Participants	(adolescen* or boy? or girl? or juvenil* or underage* or “under age” or teen? or teenager? or minor? or pubescen* or “young people” or “young person?” or youth* or [(“high school” or “middle school” or “secondary school” or “special education” or transfer) adj (student? or graduate?)] or pupil? or “emerging adult?” or pediatric? or paediatric?).tw. OR Middle School Students/ or High School Students/or Junior High School Students/or Special Education Students/or Transfer Students/or High School graduates/or Pediatrics/
Exposure	exp Social Media/ or Computer Games/or Digital Gaming/ or Blog/ or Electronic Communication/ or Computer Mediated Communication/ OR (“Social Media” or “Social Medium” or “Online Social Network*” or “virtual social world?” or “content communit*” or “Internet communication” or “communicating online” or “computer mediated communication” or “Internet group?” or Twitter or Snapchat or Facebook or Messenger or Youtube or Instagram or Tumblr or Reddit or Pinterest or blog? or blogging or vlog? or vlogging or weblogs or podcast? or skype or facetime or “Google talk” or Myspace or Flickr or Twitch or “instant message” or “instant messaging” or chat? or forum? or “Video game*” or “Computer game*” or Videogame* or Computergame* or “virtual gam* world?” or “World of warcraft” or “league of legends” or “Apex Legends” or PlayStation or Xbox or Nintendo).tw.
Outcome	Prosocial Behavior/ or Caring Behaviors/ or Altruism/ or Cooperation/ or “Assistance (Social Behavior)”/ or “Sharing (Social Behavior)”/ or “Trust (Social Behavior)”/ OR ([(prosocial or “pro social” or prosocially or “pro socially”) adj1 (behavio?r? or behave? or behaving or value? or interaction? or motivation? or “moral reasoning”)] or [(“positive online” or caring or sharing or comforting or helping or cooperative or respectful or trust*) adj (behavio?r? or interaction?)] or altruis* or helpfulness).tw.
Limit by	yr=“2014-Current” AND to (Danish or English or Norwegian or Swedish)

*This is an example of the search strategy used for PsycInfo. Search strategies were adapted to fit different search engines*.

### Eligibility Criteria

The following eligibility criteria were developed to ensure that the search and selection process returned studies of interest.

- Inclusion:
  - Participants: Age 13–18
  - Exposure: Measurement of SoMe use
  - Outcome: OPB
  - Studies published in peer-reviewed journals with full text available in English, Swedish, Danish or Norwegian from 2014.
  - Quantitative, non-experimental studies reporting on the relationship between the exposure variable and the outcome variable.
- Exclusion
  - Social media use is not covered by Kietzmann et al.'s definition (Kietzmann et al., 2011, p. 1).
  - Online prosocial behaviors are not covered by the definition by Erreygers et al. (2018a), thus excluding voluntarism and digital donations to organizations among others.

### Data Extraction

All papers from the automated database search were collated using the Rayyan Systematic Reviews web app (Ouzzani et al., 2016). After duplicates were deleted, screening was conducted to ensure that studies fulfilled the eligibility criteria. The following information was extracted from each included study:

1. Bibliography
  - Author(s)
  - Title
  - Journal
  - Year of publication
2. Study characteristics
  - Study design
  - Study setting
  - Country of origin
  - Number and age of participants
  - Gender distribution
  - The main aim of the study
  - How SoMe was defined and assessed
  - How OPB was defined and assessed
  - Type of scales used
  - Data analysis methodology
3. Results
  - Main findings

### Risk of Bias in Individual Studies

For the assessment of the risk of bias in individual studies, the Newcastle-Ottawa Scale (NOS) (Wells et al., 2014) adapted for cross-sectional studies (Herzog et al., 2013) was used. The adapted NOS uses a star system, whereby five stars are allocated for selection—two for comparability and three for outcomes (Herzog et al., 2013; Modesti et al., 2016). Thus, an article can receive a total of 10 stars, depending on the quality and the risk of bias in the article. We used the same star evaluation as (Herzog et al., 2013), divided into four groups: very good studies (9–10 points), good studies (7–8 points), satisfactory studies (5–6 points), and unsatisfactory studies (0–4 points; see Appendix C for a detailed account of the elements in the NOS adapted for cross-sectional studies). The risk of bias assessment was ascertained jointly by two of the authors (JCS and CL).

### Updated Literature Search

The literature search was updated on May 5, 2021. This update covered the period from the last search, December 9 and 10, 2019 to May 5. Some of the included databases do not allow for delimiting the search by months (e.g., Web of Science), for these databases the lower limit was set to the year 2019.

## Results

### From the Original Literature Search

The search in PsycInfo (*n* = 77), Ovid MEDLINE (R) (*n* = 70), Embase (*n* = 35), Cochrane (*n* = 9), Web of Science (*n* = 160), Sociological Abstracts and Sociological Services Abstracts (*n* = 6), and Eric (*n* = 20) resulted in 377 articles. Duplicates were deleted manually in the Endnote library, resulting in 295 unique articles. Two independent reviewers (JS and CL) conducted a blinded screening of title and abstract based on general relevancy concerning quantitative studies on SoMe and prosocial behavior. The reviewers agreed on 283 of 295 articles, yielding a total agreement score of 95.6%. The remaining 12 articles of disagreement were reviewed by a third reviewer (GJH) and discussed to reach confidence in exclusion and inclusion criteria. Primary screening and secondary reviewing and discussion excluded in total 276 articles.

**Figure 1:**
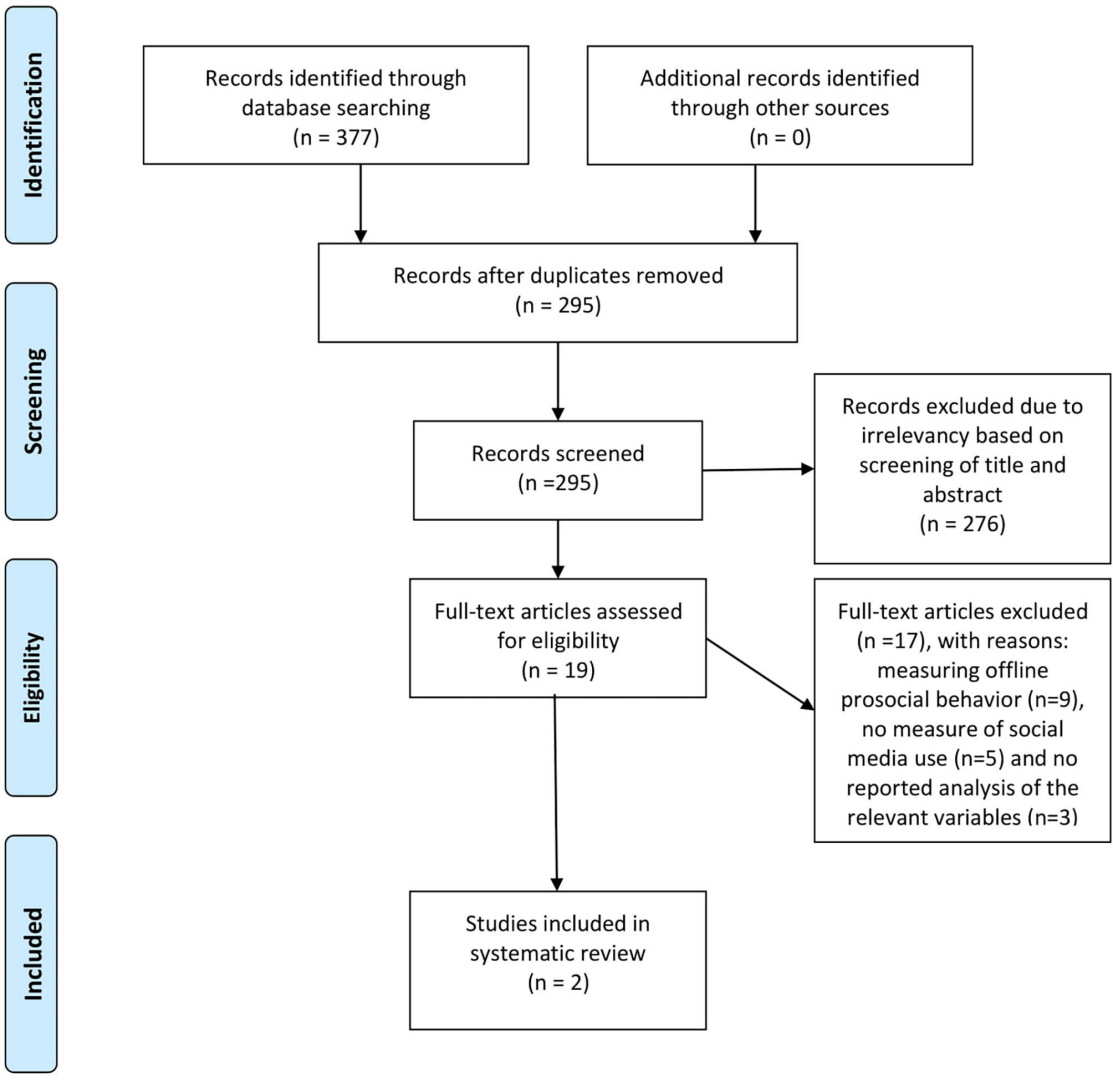
Flow diagram of the search process. PRISMA 2009.

Thus, 19 articles (Coyne et al., 2014; Prot et al., 2014; Loparev, 2016; Lu et al., 2016; Ranney, 2016; Wartberg et al., 2016; Erreygers et al., 2017, 2018b, 2019; Jin and Li, 2017; Lee et al., 2017; Greer, 2018; Guo et al., 2018; Lane and Dal Cin, 2018; Machackova et al., 2018; Meeus et al., 2018; Wang and Xing, 2018; Parlangeli et al., 2019; Lee, 2020) were assessed for eligibility based on full texts based on the original literature search. Seventeen articles were evaluated as not fulfilling the inclusion criteria due to measuring offline prosocial behavior instead of OPB (Coyne et al., 2014; Prot et al., 2014; Wartberg et al., 2016; Jin and Li, 2017; Lee et al., 2017; Greer, 2018; Lane and Dal Cin, 2018; Meeus et al., 2018; Wang and Xing, 2018; Lee, 2020), not containing measurements of SoMe use (Loparev, 2016; Lu et al., 2016; Erreygers et al., 2018b; Guo et al., 2018; Machackova et al., 2018) or not reporting any analyses or descriptive statistics on the relationship between SoMe use and OPB (Ranney, 2016; Parlangeli et al., 2019). To be clear, two of the excluded articles did include satisfactory measures of SoMe use and OPB but did not report data regarding the variables of interest or analysis of the relationship between them. See the flow chart in Figure 1.

### From the Updated Literature Search

The updated search resulted in 133 articles [PsycInfo (*N* = 17), Ovid MEDLINE (*N* = 22), Embase (*N* = 9), Cochrane (*N* = 3), Web of Science (*N* = 73), Sociological Abstracts and Sociological Services Abstracts (*N* = 1), and Eric (*N* = 8)]. Duplicates were deleted, resulting in 119 unique articles, where 24 overlapped with the original search. The remaining 95 articles were retained for assessment. Two independent reviewers (JS and CL) conducted a blinded screening of title and abstract based on general relevancy concerning quantitative studies on SoMe and prosocial behavior. The reviewers initially agreed on 94 of the 95 articles (agreement score 98.9%). One study was included after the initial assessment and reviewed in full text. The study in question did report a relevant measure of SoMe use, focused on “altruistic behaviors in social networks,” and reported a non-significant association between these factors (Zhu et al., 2020). However, the description of these behaviors seemed to be outside our definition of OPBs, covering dimensions related to “sharing with others the experiences and perceptions of their lives in social networks,” “creating a platform for a person to communicate, “sharing your successful learning experience with others in social networks,” and “network warning” (Zhu et al., 2020, p. 4). Although these dimensions may indirectly be conduit for OPB, they are not prosocial behaviors *per se*. Therefore, we decided to exclude this study.

This paper aimed to provide a quantitative assessment of the extent to which SoMe use is related to OPB among adolescents. Based on the present search, no study had the sole explicit aim to investigate the association between these variables. However, as part of a study design and/or several measures, four studies (Ranney, 2016; Erreygers et al., 2017, 2019; Parlangeli et al., 2019) measured SoMe use and OPB among adolescents. Only two of these (64 and 65) reported data on the relationship between the variables. The two included studies are authored by the same researchers. Erreygers et al. (2017) were published in the journal “*Media Psychology*” and Erreygers et al. (2019) were published in “*Journal of Happiness Studies*.” For a summary of the results, see Table 2.

**Table 2:** Data extraction of included studies.

**References**	**Title and journal**	**Study design, setting and country**	**Main aim**	**Participants**	**Type of SM and type of measure**	**Type of OPB and type of measure**	**DAM**	**Type of scales**	**Findings**
Erreygers et al. (2017)	Nice or Naughty? The Role of Emotions and Digital Media Use in Explaining Adolescents' Online Prosocial and Antisocial Behavior. In *Media Psychology*.	Cross-sectional. School. Belgium.	Examine dimensions of online prosocial and antisocial behavior and how these are related to adolescents' experienced emotions and their uses of digital media.	*N* = 1,720 (*M*age= 13.61, SD= 0.49) Boys = 784 Girls = 930 Six participants did not report on gender.	Internet use (social media, online gaming and functional media) Self-report	Performing and receiving OPB, including cheering up, comforting and supporting others. Self-report	SEM on the association between OPB and emotions, where SM was used as a mediation variable	The Online Prosocial Behavior Scale (Erreygers et al., 2018a). SM: adapted version of the EU Kids Online (2014) questionnaire for internet use.	Gaming was related negatively to performing (*b* = −0.217, *p* < 0.001) and receiving (*b* = −0.252, *p* < 0.001) OPB. Using social and audiovisual media was strongly positively associated with performing and receiving OPB (POPB: *b* = 0.768, *p* < 0.001; ROPB: *b* = 0.956, *p* < 0.001).
Erreygers et al. (2019)	Feel Good, Do Good Online? Spillover and Crossover Effects of Happiness on Adolescents' Online Prosocial Behavior. In *Journal of Happiness Studies*.	Cross-sectional and repeated measures design. Home. Belgium.	Spillover (context) and crossover (person) effects of adolescents' and their parents' daily happiness on adolescents' online prosocial behavior via a daily diary.	*N* = 136 (*M*age= 13.51, SD 0.63) Boys = 67 Girls = 69	Use of digital technologies for interpersonal contact (use of social network sites, instant messaging, emailing, texting) Self-report	Cheering up, helping, comforting and supporting via mobile phone/internet Self-report	A 1-1-1 MSEM with fixed slopes to test mediation model of T1 happiness prediction T2 OPB via T2 happiness. SM as a control variable.	OPB: 5 items based on the Online Prosocial Behavior Scale (Erreygers et al., 2018a). SM use: 5 point Likert scale on digital use	A significant positive correlation (0.39 = *p* < 0.001) between online prosocial behavior and the use of digital technologies.

*M, Mean; SD, Standard deviation; OPB, Online prosocial behavior; SM, Social media (use); DAM, Data analysis methodology; SEM, Structural equation model; MSEM, Multilevel structural equation model; T1, time 1 (after school/work); T2, time 2 (at adolescent bedtime); POBP, performing online prosocial behavior; ROPB, receiving online prosocial behavior*.

### Study Characteristics

#### Participants and Samples

The mean age for the participants in the included studies was 13.5 (Erreygers et al., 2019) and 13.6 (Erreygers et al., 2017). The sample sizes were 136 (Erreygers et al., 2019) and 1,720 (Erreygers et al., 2017). The samples contained slightly more girls than boys, with 54% (Erreygers et al., 2017) and 51% (Erreygers et al., 2019). Erreygers et al. (2017) recruited participants through schools whereas Erreygers et al. (2019) used schools, universities, SoMe, and a market research agency as recruitment arenas. Both studies were carried out in Belgium.

#### Aims, Study Design, and Measures of the Included Studies

The aims and designs of the included studies differed. Erreygers et al. (2017) aimed to investigate dimensions of online antisocial and prosocial behavior and how these were related to experienced emotions of adolescent and their use of digital media. To do so, the study used a cross-sectional design, obtaining several measures of the same population at a specific point in time. Erreygers et al. (2019) wanted to investigate spillover (context) and crossover (person) effects of adolescents' and their parents' daily happiness on adolescents' OPB via a daily diary. Spillover effects refer to the transmission of emotional states from one context (e.g., school) to another context (e.g., home) within individuals. Crossover effects refer to the transmission of emotional states between individuals. The study used a repeated-measures design via a daily diary, obtaining data on parental and adolescent happiness after school/work and in the evening, and adolescent OPB in the evening. The study also included SoMe use as a control variable as previous studies had indicated that SoMe could be a confounder in the association between happiness and OPB.

Both studies collected data using self-report measures. Erreygers et al. (2017) collected data on these outcomes once at participants' school and Erreygers et al. (2019) collected data once every evening over a period of 5 days. SoMe use was defined and measured somewhat differently. Erreygers et al. (2019) measured the “use of digital technologies for interpersonal contact,” such as the use of social networking sites, instant messaging, and sending e-mails and texts. Erreygers et al. (2017) measured “internet use.” The study used a version of the EU Kids Online questionnaire for internet use that included 11 internet activities. Although the scale was adapted for Erreygers et al. (2017), the original version has been revised and validated as part of a research toolkit used by the EU Kids Online network funded by the EC (DG Information Society) Safer Internet Program (project code SIP-KEP-321803). To explore their adapted version, the researchers ran an exploratory factor analysis. The questionnaire yielded three factors: one related to online gaming (i.e., playing online games with others), one related to the use of social and audiovisual media (i.e., visiting a social network site), and one related to the functional use of digital media (i.e., sending or receiving an email).

The two studies used similar assessments of OPB. Erreygers et al. (2017) assessed OPB as part of a larger scale including online antisocial behavior (OAB). The scale included 14 OPB elements and 11 OAB elements. The frequency of these behaviors as both the performer and the receiver was assessed. The OPB part of the scale consisted of five items adapted from the scale used by Wright and Li (2011) (i.e., “cheering up,” “offer help,” “say nice thing,” and “let someone know I care about them”) and nine adapted items from two scales (Caprara and Pastorelli, 1993; Carlo and Randall, 2002). Two of the items were poorly understood by the participants and thus not included in the final analysis, yielding a total of 12 items. The scale was later validated using the same sample, measuring the participants a second time. Exploratory factor analysis yielded 10 items, as two of the items were omitted due to low factor loadings compared to the rest of the items. The authors named the scale the Online Prosocial Behavior Scale (OPBS) (Erreygers et al., 2018a). Erreygers et al. (2019) assessed OPB using a shortened and modified version of the OPBS for diary use, leaving five items.

### Data Analysis Methodology

Erreygers et al. (2019) used a time-based daily diary design. Participants were assessed two times a day on happiness, and once a day on SoMe use and OPB. The study used a 1-1-1 multilevel structural equation model (MSEM) with fixed slopes to test the mediation model of T1 happiness predicting T2 OPB via T2 happiness (T1 = after school/work, T2 = adolescent bedtime). For the association between OPB and SoMe use, SoMe use (use of digital technologies) was used as a control variable in the MSEM for both within- and between-persons. Erreygers et al. (2017) measured OPB, emotions, and SoMe use in a standard cross-sectional design. In their main analysis, a structural equation model for the association between online behaviors and emotions was estimated. In a *post-hoc* analysis, a structural model with SoMe was used as a mediation variable between online behavior and emotions was estimated.

### Association Between Exposure and Outcome

Both studies reported a significant association between the use of SoMe and OPB. Erreygers et al. (2017) found that online gaming and using audiovisual and SoMe were related to OPB. Online gaming was related negatively to performing (*b* = −0.217, *p* < 0.001) and receiving (*b* = −0.252, *p* < 0.001) OPB, whereas using audiovisual and SoMe was strongly positively associated with performing (*b* = 0.768, *p* < 0.001) and receiving (b = 0.956, *p* < 0.001) OPB. Erreygers et al. (2019) found that the use of digital media (UDT) by adolescents was positively correlated with (performing) OPB (pOPB, UDT = 0.39, *p* < 0.001).

### Risk of Bias Assessment

Based on NOS, one study was unsatisfactory (Erreygers et al., 2019) and one was satisfactory (Erreygers et al., 2017). Erreygers et al. (2019) was considered to be at a high risk of bias. The sample size was small and unjustified, the study used convenience sampling, a non-validated self-report measure was used to measure SoMe use, and relevant confounders for the relationship between OPB and SoMe use were not adjusted for. In summary, there is a high risk of bias in the study, and one should be careful when generalizing the results. Erreygers et al. (2017) was considered to be at moderate risk of bias. Even though no sample size justification was reported, the sample size (*n* = 1,720) is considered to be more than large enough to satisfy a conservative assumption about the nature of the true population value, as long as an adequate sampling technique has been applied and the response rate is satisfactory. Random sampling was used, and the sample can be considered to be representative of the average in the target population, as 13 of 29 invited schools participated. Full information maximum likelihood (FIML) was used to estimate the model and handle missing data (Enders and Bandalos, 2001); however, the missing data were not described. The study is at risk of bias because it relies on self-report in measuring both the exposure and the outcome variable and no relevant confounders for the relationship between OPB and SoMe use were adjusted for. The study used an adapted and thus unvalidated version of a validated self-report measure to measure SoMe use. However, the scale is only slightly adapted, and at face value seems to contain the same elements as the original scale. Consequently, the use of this adapted scale will not lower the overall quality of the study.

It is important to note that neither of these studies aimed to investigate the relationship between OPB and SoMe use. Both studies included SoMe use a possible confounder or mediator. Thus, the lack of control, with regards to confounders between OPB and SoMe use, is not necessarily evidence of low study quality, because the studies did adjust for confounders with regards to the relationship between their main variables of interest. However, not controlling for confounders between OPB and SoMe use indicates a risk of bias in the results reported on that particular relationship. Consequently, the results should be approached with caution. For a summary of the risk of bias assessment, see Table 3 (for a detailed account of the risk of bias assessment, see Appendix C).

**Table 3:** Summary of risk of bias assessment.

**Criteria**	**Erreygers et al. (2019)**	**Erreygers et al. (2017)**
Representativeness of the sample	0	*
Sample size	0	*
Non-respondents	*	*-
Ascertainment of the exposure	0	*-
Comparability	0	0
Assessment of outcome	*	*
Statistical tests	*	*
Total score	***= Unsatisfactory	*****- = Satisfactory

*Star evaluation: very good studies (9–10 points), good studies (7–8 points), satisfactory studies (5–6 points), and unsatisfactory studies (0–4 points)*.

### Strategy for Data Synthesis

In the protocol (registered at PROSPERO), a strategy for data synthesis was described. The plan was to conduct a meta-analysis to estimate the overall association between the use of SoMe and OPB. This step was, however, only deemed viable if at least four studies were included in the review and dependent upon a similar enough design of the included studies (low heterogeneity). If a meta-analysis was not deemed possible, we planned to summarize the evidence in a narrative style. As only two studies fulfilled the eligibility criteria, a meta-analysis was not conducted and only a narrative summary of the evidence is given in the present study.

## Discussion

This paper aimed to examine to what extent SoMe use is related to OPB among adolescents. A review of primary studies on that relationship was conducted using a systematic and transparent approach and resulted in two studies, which met the eligibility criteria.

Although both studies included in this review reported an association between SoMe use and OPB among adolescents; it is clear that the amount of quantitative data and studies on the present relationship is scarce. In addition, the quality of the present data may not be adequate. Consequently, associations cannot be established based on the current research. However, some points from these articles will be discussed, which may aid future research directions on the topic.

### The Relationship Between SoMe Use and OPB Among Adolescents

In this review, OPB was defined as voluntary behavior carried out in an electronic/SoMe context with the intention of benefitting particular others or promoting harmonious relations with others. Erreygers et al. (2017) and Erreygers et al. (2019) reported an association between SoMe use and OPB among adolescents. In other words, the more adolescents use SoMe, the more OPB they display. Studies not included in this review due to time of publication (Lister, 2007) or focusing on another age group (Wright and Li, 2011; young adults, Kinnunen et al., 2016; adults) also support the association between SoMe use and OPB. More specifically, Lister (2007) found an association between computer-mediated communication, defined as instant messaging and visiting SoMe sites (coined as “blogging”), and OPBs among American adolescents in 7th grade (12–13 years), 9th grade (14–15 years), and 11th grade (16–17 years). Wright and Li (2011) found that time spent using electronic technologies was correlated with OPB through that particular technology, such as social networking sites, chat programs, email, and text-messages, among young adults (mean age = 20 years). Kinnunen et al. (2016) found that the use of SoMe, defined as time spent on different SoMe sites such as Facebook, YouTube, and Wikipedia, was associated with help-giving and moral courage among university students in Finland (mean age = 26 years). These studies did not fulfill the eligibility criteria and thus were not included in the review. However, they do serve as corroboratory evidence of a possible association between SoMe and OPB.

Erreygers et al. (2017) reported different associations for different subtypes of SoMe use. The authors reported a positive association between OPB and audiovisual and SoMe (i.e., visiting a social network site or vlogging site), a negative association with gaming (i.e., playing online games with others), and no significant relationship with the functional use of digital media (i.e., sending or receiving an email). These results are supported by prior research from Wright and Li (2011) who found a stronger positive association between chat programs and social networks and OPB, than between e-mails and text messages and OPB, among young adults. In other words, young adults seem to be engaging more in OPB when visiting a social network site or vlogging site (i.e., YouTube), than when they send text messages or e-mails. In sum, these studies indicate that different forms of SoMe may relate to OPB in different ways. “Classic SoMe,” such as social network sites, may be positively correlated with OPB, while functional use of SoMe may be weekly correlated or not correlated with OPB, and online gaming may be negatively correlated with OPB.

Erreygers et al. (2017) measured both receiving and performing OPB, finding associations with audiovisual and SoMe use for both variables of OPB. In other words, the more adolescents visited SoMe or used audiovisual media, the more prosocially they behaved online and the more they received prosocial reactions from others. Drawing from research that indicates an association between prosocial media content and prosocial behavior (Coyne et al., 2018), it is plausible that consuming positive audiovisual media content and messages could elicit OPBs, which could, in turn, elicit prosocial reactions from peers.

### The Quality of the Data in the Present Review

Erreygers et al. (2017) and Erreygers et al. (2019) found notable associations between SoMe and OPB. Erreygers et al. (2017) also indicated differences in the relationships between OPB and typical SoMe (i.e., social networking sites) and OPB and online gaming. Studies not included in this review due to being published prior to 2014 (Lister, 2007) and focusing on adults (Wright and Li, 2011; Kinnunen et al., 2016) support these findings.

Although these results are interesting, they are not enough to establish associations. First, neither Erreygers et al. (2017) nor Erreygers et al. (2019) controlled for confounding variables, thereby making it difficult to eliminate alternative explanations. For example, some studies have indicated gender differences in adolescent (offline) prosocial behavior (Eisenberg et al., 2001; Caravita et al., 2009; Van der Graaff et al., 2018). Moreover, Lister (2007) found that females reported a higher degree of OPB than males. However, Wright and Li (2011) and Wang and Wang (2008) found no gender differences in OPB. With the effects of gender remaining unclear, controlling for gender as a possible confounder in the SoMe use–OPB relationship would be beneficial.

Research on offline prosocial behavior among adolescents and children indicates several possible relevant confounding variables. Studies show a decline in prosocial behaviors during early and middle adolescence (Carlo et al., 2007; Kanacri et al., 2013; Jambon and Smetana, 2014), suggesting age as a relevant confounder. Personality has also been shown to strongly predict prosocial behaviors among adolescents, especially morally relevant personality traits and resiliency (Padilla-Walker and Fraser, 2014; Xie et al., 2016). Some studies have indicated significant links between socio-economic status (SES) and prosocial behavior (Eisenberg et al., 2007). Prosocial behavior in rural areas may be relatively low due to depleted social capital and community resources (Carlo et al., 2007), compared to adolescents from more urban areas and middle-to-high SES families (Van der Graaff et al., 2018). However, one large study (Plenty et al., 2015) indicated the importance of the school environment showing that students who experience more manageable school demands and social support from teachers and classmates are more likely to display more prosocial behaviors. Thus, both SES and school environment could be important confounders. Lastly, the recipient of prosocial behavior may be a relevant confounding factor, as evidence indicate that prosocial behaviors in adolescence increase toward friends, but not toward members of one‘s family (Padilla-Walker et al., 2013).

Second, the assessment of the risk of bias in the included studies revealed that Erreygers et al. (2019) were unsatisfactory and thus at a high risk of bias and that Erreygers et al. (2017) were barely satisfactory and thus with a moderate risk of bias. One of the reasons for this is the use of self-report measures in both the studies. Although highly cost-effective, self-report measures are at high risk of social desirability bias, especially relevant when measuring OPB. Social desirability can be defined as the tendency for research subjects to give answers, which will be viewed favorably by others, instead of responses reflecting their true feelings. It can take the form of overreporting “good behavior,” underreporting “bad behavior,” or a combination of both. Research shows that social desirability influences the results in almost half of all studies using self-report (Van de Mortel, 2008). Social desirability scales can be used to limit the effects of social desirability, however, neither of the studies in this review did so.

In addition, self-report methods in relation to SoMe use have demonstrated low-to-moderate correlations with actual use, when comparing self-reports and tracking data. This has been shown when measuring both internet use (Scharkow, 2016; Araujo et al., 2017) and social network use (Junco, 2013; Scharkow, 2016). The typical tendency is overreporting (Araujo et al., 2017).

Third, although the included studies in this review (Erreygers et al., 2017, 2019) used a validated instrument of OPB, the OPBS is a global measure of OPB. Global measures of prosocial behavior have been criticized (Padilla-Walker and Carlo, 2015; Coyne et al., 2018). The vast research base on (offline) prosocial behaviors has shown that prosocial behaviors differ in their motivations, and hence in social and psychological outcomes (see Padilla-Walker and Carlo, 2015 for a detailed account). For example, Carlo et al. (2010) found evidence for six different prosocial behaviors. The limitations of using a global measure of OPB may be numerous, but the most pressing limitation concerns the validity of the results derived from the global measures. It may be the case that one of several subtypes of OPB (e.g., helping vs. sharing or altruistically motivated vs. egotistically motivated prosocial behavior) can explain much of the variance in the OPB-SM use relationship. The researchers behind the OPBS themselves encourage the development of a more elaborate measure of OPB covering different subtypes (Erreygers et al., 2018a).

Fourth, the studies included in this review (Erreygers et al., 2017, 2019) contained similar groups of participants in terms of culture. The participants were all Belgian adolescents and thus generalizing the findings to other countries and cultures may not be warranted yet. The researchers note the need for more diversity in the samples, in terms of nationality and culture, to corroborate their results. This point is substantiated by the aforementioned research on the links between (offline) prosocial behavior and SES.

### Scarcity of Eligible Studies

The present review reveals a paucity of studies related to the use of SoMe and OPB. Only four studies (Ranney, 2016; Erreygers et al., 2017, 2019; Parlangeli et al., 2019) that measured SoMe use and OPB among adolescents were identified. Further, only two of these (Erreygers et al., 2017, 2019) reported relevant data on the relationship between the variables of interest.

Two possible explanations for the scarcity of eligible studies emerge. Firstly, the eligibility criteria may have been too narrow. The criteria demanded quantitative studies reporting adequate data on the relationship between OPB and SoMe use among adolescents (13–18 years), published between 2014 and 2019. Wright and Li (2012) did refer to a number of qualitative studies on OPB in 2012, which may indicate a substantial qualitative research base on OPB, considering the increase in research concerning digital media. However, this research was deemed to be outside the scope of this review focusing on the quantitative association between SoMe use and OPB.

To investigate if more articles of relevance could be found by loosening the criteria, a thorough hand search and *snowballing* (i.e., reading articles cited in articles identified in the present review) was conducted. This search was only focused on studies containing relevant data on the relationship between SoMe use and OPB. The investigation revealed no additional articles which met the original eligibility criteria and resulted in only three studies containing relevant data on SoMe use and OPB, although in different/unwanted target groups (Wright and Li, 2011; Kinnunen et al., 2016) or which was published prior to 2014 (Lister, 2007; Wright and Li, 2011). There are therefore no strong indications that the strict eligibility criteria were mainly responsible for the low number of included studies.

Thus, the other possible explanation does not concern the eligibility criteria, but a scarcity of OPB studies in general. There is a vast base of research on media and media effects on children and adolescents (Valkenburg et al., 2021). However, some researchers (Livingstone, 1996; de Leeuw and Buijzen, 2016) note that media research traditionally has contained an imbalance in research attention. More specifically, there seems to be a bias in research attention regarding “bad content” and negative effects of media compared to positive content and positive effects. Recent studies have, however, also focused on the potential positive aspects of SoMe use (de Leeuw and Buijzen, 2016; Scott et al., 2019; Thomas et al., 2020; Hjetland et al., 2021)—for instance, recognizing how SoMe play a key role in the social lives of adolescents (Hjetland et al., 2021).

As a related issue, Erreygers et al. (2017) note the seemingly paradoxical fact that the amount of research devoted to antisocial online behavior (AOB) vs. OPB is almost opposite to the actual occurrence of this behavior. In their study, they investigated the simultaneous occurrence of AOB next to OPB and found that OPBs were much more prevalent. Those findings are supported by Lister (2007), which also found that OPBs were more prevalent than AOB. de Leeuw and Buijzen (2016) stressed the importance of balancing the research on positive and negative behavior and effects of (social) media, as there are enormous potentials for child and youth development to be explored in media, in particular SoMe.

### Strengths and Limitations

A primary strength of the present study is the use of standardized guidelines to carry out a review in a transparent and robust way. It is also to the best of our knowledge the first review to investigate this area within the field of adolescent SoMe use. Finally, an additional updated literature search was performed in May 2021, which increases the likelihood of identifying any new developments within the scope of the present study.

The present review also has some limitations. First, the search may not have covered all relevant literature, and only included published peer-reviewed papers (i.e., not gray literature). Even though SoMe use was widely operationalized, the way in which OPB was operationalized may have excluded some relevant articles. “OPB” as a term is fairly new and may not necessarily be the nomenclature used in fields outside psychology or social sciences. Other kinds of OPBs were also outside the scope of this review, and online prosociality in the form of civic engagement, voluntary work, and donations to organizations have merit in their own right. This would be an interesting avenue to investigate for future reviews. However, the stringent search with well-defined search criteria is one of the strengths of this review. The search was developed in collaboration with specialist librarians at the Norwegian Institute for Public Health, test search studies were conducted prior to the main search in order to increase sensitivity and specificity, and the search covered seven large databases in social, psychological, and health sciences.

Second, there were too few studies included in this review to establish an association and to conduct a meta-analysis. However, finding only two studies that fulfilled the eligibility criteria is a finding in itself, and as we have argued in the sections above, seems to be indicative of a research gap within the field.

Third, the search had a lower limit of papers published in 2014. This decision was based on the rapid changes in the use and type of SoMe platforms available. Findings more than 5 years old were therefore deemed to be less relevant to shed light on the contemporary association between SoMe use and OPBs.

Finally, although the NOS adapted for cross-sectional studies has proven to be quick, adaptable, and to show moderate reliability, compared to the widely used Appraisal Tool for Cross-Sectional Studies (AXIS) (Moskalewicz and Oremus, 2020), it has not been thoroughly validated. It has merely been adapted for the use of cross-sectional studies, without thorough testing and validation. Therefore, even though the risk of bias assessment in this review was thoroughly conducted, the use of NOS may have unintentionally skewed the risk of bias assessment in either a low-risk or a high-risk direction.

## Conclusions and Future Directions

The present review included two studies that met the eligibility criteria. Although both studies found an association between OPB and SoMe use among adolescents, the results are not strong enough to establish an association. Finding only two studies indicates a research gap in the field and additional research on the subject is required and warranted. To aid future research on the subject, the next section will propose possible topics of inquiry.

First, future research on the relationship between OPB and SoMe use may benefit from looking at different subtypes of SoMe in relation to OPB. It may be that functional media use, social networking and vlogging, and online gaming all relate to OPB in different ways. It would also be particularly interesting to investigate whether the negative correlation between OPB and online gaming found in Erreygers et al. (2017) could be mediated by gaming content (i.e., prosocial vs. antisocial content).

Second, future studies may benefit from including potential confounders and moderators when investigating the relationship between OPB and SoMe use, such as gender, age, personality types, SES, school environment, and the recipient of the behavior (i.e., directed at friend vs. family).

Third, to increase the validity of and accuracy in the data collected, future studies could benefit from including social desirability scales (Van de Mortel, 2008) in relation to OPB and match tracking data with self-reports in relation to SoMe use. Finally, offline prosocial behavior is considered to be a multidimensional construct (Padilla-Walker and Carlo, 2015), which eludes to the limited usefulness of a global measure of prosocial behavior. Thus, there are ample reasons to view its online counterpart as a multidimensional construct as well. Consequently, future research could benefit from looking at prosocial behavior and its subtypes (i.e., altruistically and egotistically motived prosocial behavior). Although, it should be noted that the subtypes of OPB could be quite different from the subtypes of offline prosocial behavior (i.e., online donations, online activism, and online sharing).

## Author Contributions

JCS and CL conceptualized the aim and designed the paper, collected the data, and reviewed the included studies. CL drafted the first version of the paper in collaboration with JCS, GJH, and TB. GJH acted to solve any conflict related to the selection of the studies. All authors contributed to further revisions of the paper. All authors contributed to the revisions of the paper after peer-review, and have approved the final version of this paper.

## Funding

The open access publication fee has been funded by University of Bergen.

## Conflict of Interest

The authors declare that the research was conducted in the absence of any commercial or financial relationships that could be construed as a potential conflict of interest.

## Publisher's Note

All claims expressed in this article are solely those of the authors and do not necessarily represent those of their affiliated organizations, or those of the publisher, the editors and the reviewers. Any product that may be evaluated in this article, or claim that may be made by its manufacturer, is not guaranteed or endorsed by the publisher.

## Footnotes

^1^Apple Reinvents the Phone with iPhone. Apple Inc. (2007).
