# Supplementary material for: A Review of the Relationship Between Social Media Use and Online Prosocial Behavior Among Adolescents
Source: Front Psychol. 2021 Sep 28;12:579347. doi: 10.3389/fpsyg.2021.579347 (PMC8506009; doi:10.3389/fpsyg.2021.579347)
Supplement: Supplementary file 2 [file Table_2.pdf]

Search strategy – all databases

Used both in the original search in December 2019 and in the updated literature search in May 2021.

Contents

Database: PsycINFO.. 2

Database: Ovid MEDLINE(R) and Epub Ahead of Print, In-Process & Other Non-Indexed Citations, Daily and Versions(R)... 4

Database: Embase 5

Database: Cochrane Database of Systematic Reviews, Central Register of Controlled trials 6

Database: Web of Science 7

Database: Sociological Abstracts (1952 – current) og Sociological Services Abstracts 9

Database: Eric 11

Database: PsycINFO

1	Prosocial Behavior/ or Caring Behaviors/ or Altruism/ or Cooperation/ or "Assistance (Social Behavior)"/ or "Sharing (Social Behavior)"/ or "Trust (Social Behavior)"/	40507
2	(((prosocial or "pro social" or prosocially or "pro socially") adj1 (behavio?r? or behave? or behaving or value? or interaction? or motivation? or "moral reasoning")) or (("positive online" or caring or sharing or comforting or helping or cooperative or respectful or trust*) adj (behavio?r? or interaction?)) or altruis* or helpfulness).tw.	22888
3	1 or 2	52330
4	exp Social Media/ or Computer Games/ or Digital Gaming/ or Blog/ or Electronic Communication/ or Computer Mediated Communication/	28758
5	("Social Media" or "Social Medium" or "Online Social Network*" or "virtual social world?" or "content communit*" or "Internet communication" or "communicating online" or "computer mediated communication" or "Internet group?" or Twitter or Snapchat or Facebook or Messenger or Youtube or Instagram or Tumblr or Reddit or Pinterest or blog? or blogging or vlog? or vlogging or weblogs or podcast? or skype or facetime or "Google talk" or Myspace or Flickr or Twitch or "instant message" or "instant messaging" or chat? or forum? or "Video game*" or "Computer game*" or Videogame* or Computergame* or "virtual gam* world?" or "World of warcraft" or "league of legends" or "Apex Legends" or PlayStation or Xbox or Nintendo).tw.	44242
6	4 or 5	54785
7	Middle School Students/ or High School Students/ or Junior High School Students/ or Special Education Students/ or Transfer Students/ or High School graduates/ or Pediatrics/	77717
8	(adolescen* or boy? or girl? or juvenil* or underage* or "under age" or teen? or teenager? or minor? or pubescen* or "young people" or "young person?" or youth* or (("high school" or "middle school" or "secondary school" or "special education" or transfer) adj (student? or graduate?)) or pupil? or "emerging adult?" or pediatric? or paediatric?).tw.	508544
9	7 or 8	531663
10	3 and 6 and 9	134
11	limit 10 to yr="2014 -Current"	77

12	limit 11 to (danish or english or norwegian or swedish)	77

Database: Ovid MEDLINE(R) and Epub Ahead of Print, In-Process & Other Non-Indexed Citations, Daily and Versions(R)

1	Helping behavior/ or Altruism/ or Cooperative behavior/	51108
2	(((prosocial or "pro social" or prosocially or "pro socially") adj1 (behavio?r? or behave? or behaving or value? or interaction? or motivation? or "moral reasoning")) or (("positive online" or caring or sharing or comforting or helping or cooperative or respectful or trust*) adj (behavio?r? or interaction?)) or altruis* or helpfulness).tw,kf.	14988
3	1 or 2	62899
4	Online social networking/ or Social media/ or Video games/ or Blogging/	12461
5	("Social Media" or "Social Medium" or "Online Social Network*" or "virtual social world?" or "content communit*" or "Internet communication" or "communicating online" or "computer mediated communication" or "Internet group?" or Twitter or Snapchat or Facebook or Messenger or Youtube or Instagram or Tumblr or Reddit or Pinterest or blog? or blogging or vlog? or vlogging or weblogs or podcast? or skype or facetime or "Google talk" or Myspace or Flickr or Twitch or "instant message" or "instant messaging?" or chat? or forum? or "Video game*" or "Computer game*" or Videogame* or Computergame* or "virtual gam* world?" or "World of warcraft" or "League of Legends" or "Apex Legends" or PlayStation or Xbox or Nintendo).tw,kf.	121598
6	4 or 5	125801
7	Pediatrics/ or Adolescent/	2018336
8	(adolescen* or boy? or girl? or juvenil* or underage* or "under age" or teen? or teenager? or minor? or pubescen* or "young people" or "young person?" or youth* or (("high school" or "middle school" or "secondary school" or "special education" or transfer) adj (student? or graduate?)) or pupil? or "emerging adult?" or pediatric? or paediatric?).tw,kf.	1128640
9	7 or 8	2688711
10	3 and 6 and 9	130
11	limit 10 to (yr="2014 -Current" and (danish or english or multilingual or norwegian or swedish))	70

Database: Embase

1	Altruism/ or Cooperation/	48718
2	(((prosocial or "pro social" or prosocially or "pro socially") adj1 (behavio?r? or behave? or behaving or value? or interaction? or motivation? or "moral reasoning")) or (("positive online" or caring or sharing or comforting or helping or cooperative or respectful or trust*) adj (behavio?r? or interaction?)) or altruis* or helpfulness).tw,kw.	17011
3	1 or 2	60298
4	Online social network/ or Social media/ or Video game/ or Blogging/	20739
5	("Social Media" or "Social Medium" or "Online Social Network*" or "virtual social world?" or "content communit*" or "Internet communication" or "communicating online" or "computer mediated communication" or "Internet group?" or Twitter or Snapchat or Facebook or Messenger or Youtube or Instagram or Tumblr or Reddit or Pinterest or blog? or blogging or vlog? or vlogging or weblogs or podcast? or skype or facetime or "Google talk" or Myspace or Flickr or Twitch or "instant message" or "instant messaging" or chat? or forum? or "Video game*" or "Computer game*" or Videogame* or Computergame* or "virtual gam* world?" or "World of Warcraft" or "League of Legends" or "Apex Legends" or PlayStation or Xbox or Nintendo).tw,kw.	144785
6	4 or 5	150938
7	Middle school student/ or High school student/ or High school graduate/ or Pediatrics/ or Adolescent/ or Juvenile/	1557208
8	(adolescen* or boy? or girl? or juvenil* or underage* or "under age" or teen? or teenager? or minor? or pubescen* or "young people" or "young person?" or youth* or (("high school" or "middle school" or "secondary school" or "special education" or transfer) adj (student? or graduate?)) or pupil? or "emerging adult?" or pediatric? or paediatric?).tw,kw.	1495230
9	7 or 8	2512556
10	3 and 6 and 9	135
11	limit 10 to ((conference abstracts or embase) and (danish or english or norwegian or swedish) and yr="2014 -Current")	35

Database: Cochrane Database of Systematic Reviews, Central Register of Controlled trials

#1	[mh ^"Helping behavior"]	86
#2	[mh ^"Altruism"]	89
#3	[mh ^"Cooperative behavior"]	962
#4	((("prosocial" or "pro social" or "prosocially" or "pro socially") NEAR/1 ("behavio?r?" or "behave?" or "behaving" or "value?" or "interaction?" or "motivation?" or "moral reasoning")) or (("positive online" or "caring" or "sharing" or "comforting" or "helping" or "cooperative" or "respectful" or "trust*") NEXT ("behavio?r?" or "interaction?")) or "altruis*" or "helpfulness"):ti,ab	319
#5	{OR #1-#4}	1427
#6	[mh ^"Online social networking"]	4
#7	[mh ^"Social media"]	121
#8	[mh ^"Video games"]	618
#9	[mh ^"Blogging"]	13
#10	("Social Media" or "Social Medium" or "Online Social Network*" or "virtual social world?" or "content communit*" or "Internet communication" or "communicating online" or "computer mediated communication" or "Internet group?" or Twitter or Snapchat or Facebook or Messenger or Youtube or Instagram or Tumblr or Reddit or Pinterest or blog? or blogging or vlog? or vlogging or weblogs or podcast? or skype or facetime or "Google talk" or Myspace or Flickr or Twitch or "instant message" or "instant messaging" or chat? or forum? or "Video game*" or "Computer game*" or Videogame* or Computergame* or "virtual gam* world?" or "World of Warcraft" or "League of Legends" or "Apex Legends" or PlayStation or Xbox or Nintendo):ti,ab	6101
#11	{OR #6-#10}	6415
#12	[mh ^"Pediatrics"]	609
#13	[mh ^"Adolescent"]	101821
#14	(adolescen* or boy? or girl? or juvenil* or underage* or "under age" or teen? or teenager? or minor? or pubescen* or "young people" or "young person?" or youth* or (("high school" or "middle school" or "secondary school" or "special education" or transfer) NEXT (student? or graduate?)) or pupil? or "emerging adult?" or pediatric? or paediatric?).ti,ab	3827
#15	{OR #12-#14}	105810
#16	#5 AND #11 AND #15	9

Database: Web of Science

# 6	160	#3 AND #2 AND #1<br>Refined by: PUBLICATION YEARS: (2015 OR 2019 OR 2014 OR 2018 OR 2017 OR 2016) AND LANGUAGES: (ENGLISH)<br>Indexes=SCI-EXPANDED, SSCI, A&HCI, ESCI Timespan=All years
# 5	172	#3 AND #2 AND #1<br>Refined by: PUBLICATION YEARS: (2015 OR 2019 OR 2014 OR 2018 OR 2017 OR 2016)<br>Indexes=SCI-EXPANDED, SSCI, A&HCI, ESCI Timespan=All years
# 4	235	#3 AND #2 AND #1<br>Indexes=SCI-EXPANDED, SSCI, A&HCI, ESCI Timespan=All years
# 3	1,443,768	TOPIC: (("adolescen*" OR "boy$" OR "girl$" OR "juvenil*" OR "underage*" OR "under age" OR "teen$" OR "teenager$" OR "minor$" OR "pubescen*" OR "young people" OR "young person$" OR "youth*" OR (("high school" OR "middle school" OR "secondary school" OR "special education" OR "transfer") NEAR/0 ("student$" OR "graduate$")) OR "pupil$" OR "emerging adult$" OR "pediatric$" OR "paediatric$"))<br>Indexes=SCI-EXPANDED, SSCI, A&HCI, ESCI Timespan=All years
# 2	425,947	TOPIC: (("Social Media" or "Social Medium" or "Online Social Network*" or "virtual social world$" or "content communit*" or "Internet communication" or "communicating online" or "computer mediated communication" or "Internet group$" or "Twitter" or "Snapchat" or "Facebook" or "Messenger" or "Youtube" or "Instagram" or "Tumblr" or "Reddit" or "Pinterest" or "blog$" or "blogging" or "vlog$" or "vlogging" or "weblogs" or "podcast$" or "skype" or "facetime" or "Google talk" or "Myspace" or "Flickr" or "Twitch" or "instant message" or "instant messaging" or "chat$" or "forum$" or "Video game*" or "Computer game*" or "Videogame*" or "Computergame*" or "virtual gam* world$" or "World of Warcraft" or "League of Legends" or "Apex Legends" or "PlayStation" or "Xbox" or "Nintendo"))<br>Indexes=SCI-EXPANDED, SSCI, A&HCI, ESCI Timespan=All years
# 1	33,369	TS=(((("prosocial" OR "pro social" OR "prosocially" OR "pro socially") NEAR/0 ("behavior$" OR "behaviour$" OR "behave$" OR "behaving" OR "value$" OR "interaction$" OR "motivation$" OR "moral reasoning")) OR (("positive online" OR "caring" OR "sharing" OR "comforting" OR "helping"

		OR "cooperative" OR "respectful" OR "trust*") NEAR/0 ("behavior$" OR "behaviour$" OR "interaction$")) OR "altruis*" OR "helpfulness")) Indexes=SCI-EXPANDED, SSCI, A&HCI, ESCI Timespan=All years

Database: Sociological Abstracts (1952 – current) og Sociological Services Abstracts

S1	(SU.EXACT("Helping behavior") OR SU.EXACT("Altruism")) AND la.exact("Norwegian" OR "Swedish" OR "English" OR "Danish") AND pd(>20140101)	368
S2	AB,TI(((prosocial OR "pro social" OR prosocially OR "pro socially") NEAR/0 (behavior* OR behaviour* OR behave* OR behaving OR value* OR interaction* OR motivation* OR "moral reasoning")) OR (("positive online" OR caring OR sharing OR comforting OR helping OR cooperative OR respectful OR trust*) NEAR/0 (behavior* OR behaviour* OR interaction*)) OR altruis* OR helpfulness) AND la.exact("Norwegian" OR "Swedish" OR "English" OR "Danish") AND pd(>20140101)	1156
S3	S1 or S2	1232
S4	SU.EXACT("Computer mediated communication") AND la.exact("Norwegian" OR "Swedish" OR "English" OR "Danish") AND pd(>20140101)	305
S5	AB,TI("Social Media" OR "Social Medium" OR "Online Social Network*" OR "virtual social world" OR "virtual social worlds" OR "content communit*" OR "Internet communication" OR "communicating online" OR "computer mediated communication" OR "Internet group" OR "Internet groups" OR Twitter OR Snapchat OR Facebook OR Messenger OR Youtube OR Instagram OR Tumblr OR Reddit OR Pinterest OR blog OR blogs OR blogging OR vlog OR vlogs OR vlogging OR weblogs OR podcast OR podcasts OR skype OR facetime OR "Google talk" OR Myspace OR Flickr OR Twitch OR "instant message" OR "instant messaging" OR chat* OR forum OR forums OR "Video game*" OR "Computer game*" OR Videogame* OR Computergame* OR "virtual gam* world" OR "virtual gam* worlds" OR "World of warcraft" OR "league of legends" OR "Apex Legends" OR PlayStation OR Xbox OR Nintendo) AND la.exact("Norwegian" OR "Swedish" OR "English" OR "Danish") AND pd(>20140101)	5755
S6	S4 or S5	5903
S7	(SU.EXACT("Adolescents") OR SU.EXACT("High school students") OR SU.EXACT("Junior high school students") OR SU.EXACT("Youth")) AND la.exact("Norwegian" OR "Swedish" OR "English" OR "Danish") AND pd(>20140101)	16453
S8	AB,TI(adolescen* OR boy OR boys OR girl OR girls OR juvenil* OR underage* OR "under age" OR teen OR teens OR teenager OR teenagers OR minor OR minors OR pubescen* OR "young people" OR "young person" OR "young persons" OR youth OR youths OR (("high school" OR "middle school" OR "secondary school" OR "special	26433

	education" OR transfer) PRE/0 (student OR students OR graduate OR graduates)) OR pupil OR pupils OR "emerging adult" OR "emerging adults" OR pediatric OR pediatrics OR paediatric OR paediatrics) AND la.exact("Norwegian" OR "Swedish" OR "English" OR "Danish") AND pd(>20140101)	
S9	S7 or S8	26867
S10	S3 and S6 and S9	6

Database: Eric

S10	S3 AND S6 AND S9	20
S9	S7 OR S8	35,019
S8	AB,TI(adolescen* OR boy* OR girl* OR juvenil* OR underage* OR "under age" OR teen* OR teenager* OR minor* OR pubescen* OR "young people" OR "young person*" OR youth* OR (("high school" OR "middle school" OR "secondary school" OR "special education" OR transfer) NEAR/0 (student* OR graduate*)) OR pupil* OR "emerging adult*" OR pediatric* OR paediatric*) AND la.exact("Norwegian" OR "Swedish" OR "English" OR "Danish") AND pd(>20140101)	26,893
S7	SU.EXACT("Middle School Students") OR SU.EXACT("High School Students") OR SU.EXACT("Junior High School Students") OR SU.EXACT("Special Needs Students") OR SU.EXACT("Transfer Students") OR SU.EXACT("High School Graduates") OR SU.EXACT("Pediatrics") OR SU.EXACT("Adolescents") OR SU.EXACT("Early Adolescents") OR (SU.EXACT("Late Adolescents") OR SU.EXACT("Youth")) AND la.exact("Norwegian" OR "Swedish" OR "English" OR "Danish") AND pd(>20140101)	19,717
S6	S4 OR S5	8,335
S5	AB,TI("Social Media" OR "Social Medium" OR "Online Social Network*" OR "virtual social world*" OR "content communit*" OR "Internet communication" OR "communicating online" OR "computer mediated communication" OR "Internet group*" OR Twitter OR Snapchat OR Facebook OR Messenger OR Youtube OR Instagram OR Tumblr OR Reddit OR Pinterest OR blog* OR blogging OR vlog* OR vlogging OR weblogs OR podcast* OR skype OR facetime OR "Google talk" OR Myspace OR Flickr OR Twitch OR "instant message" OR "instant messaging" OR chat* OR forum* OR "Video game*" OR "Computer game*" OR Videogame* OR Computergame* OR "virtual gam* world*" OR "World of Warcraft" OR "League of Legends" OR "Apex Legends" OR PlayStation OR Xbox OR Nintendo) AND la.exact("Norwegian" OR "Swedish" OR "English" OR "Danish") AND pd(>20140101)	5,322
S4	SU.EXACT("Social Media") OR SU.EXACT("Computer Games") OR SU.EXACT("Video Games") OR SU.EXACT("Computer Mediated Communication")	5,808
S3	S1 OR S2	2,661
S2	AB,TI(((prosocial OR "pro social" OR prosocially OR "pro socially") NEAR/0 (behavior* OR behaviour* OR behave* OR behaving OR value* OR interaction* OR motivation? OR "moral reasoning")) OR (("positive online" OR caring OR sharing OR comforting OR helping OR cooperative OR respectful OR trust*) NEAR/0 (behavior* OR behaviour* OR interaction*)) OR altruis* OR helpfulness) AND la.exact("Norwegian" OR "Swedish" OR "English" OR "Danish") AND pd(>20140101)	677
S1	SU.EXACT("Prosocial Behavior") OR SU.EXACT("Helping Relationship") OR SU.EXACT("Altruism") OR SU.EXACT("Trust (Psychology)")	2,391
