# Supplementary material for: A Review of the Relationship Between Social Media Use and Online Prosocial Behavior Among Adolescents
Source: Front Psychol. 2021 Sep 28;12:579347. doi: 10.3389/fpsyg.2021.579347 (PMC8506009; doi:10.3389/fpsyg.2021.579347)
Supplement: Supplementary file 3 [file Table_3.pdf]

Appendix C

A detailed description of the risk of bias assessment

Criteria	**Assesment**	**With reference to:**
**Erreygers, Vandebosch (65)**		
Representativeness of the sample	Convenience sampling	Quote: "*36 families were recruited by the first two authors via four secondary schools, two universities, and social media … 100 additional families were recruited via a market research agency*"<br>Comment: eligibility criteria for participation in the study were strict. However, no report of random sampling technique or estimation of representativeness to the target group.
Sample size	Small sample size and sample size justification not reported	Quote: "*The participants were 136 adolescents (67 boys, 69 girls) and 234 working parents"* and *"our relatively small sample size limited statistical power, which precluded including other possible relevant control variables*".<br>Comment: whether the sample size is based on calculations is not described.
Non-respondents	Basic summary of non-respondent characteristics in sampling frame is described *****	Quote: "*resulted in the following missing data rates: 22 (3.2%) of the adolescents', 8 (1.2%) of the mothers', and 7 (1.4%) of the fathers' entries at T1; and 51 (7.5%) of the adolescents' entries at T2*".<br>Comment: there are few missing data and adequate measures for handling of the missing data were reported.
Ascertainment of the exposure	Use of unvalidated self-report measure constructed by the researchers.	Quote: "*use of digital technologies for interpersonal contact (e.g., use of social network sites, instant messaging, emailing, texting) throughout the day, on a 5-point Likert-type scale*"
Comparability	Not controlled for confounders/level of adjustment	Comment: the main aim of the study was not to investigate the SM-OPB relationship, and the variable of SM use in itself was included due to it being a possible confounder. Therefore, not adjusting for possible confounders in the association between SM and OPB is not evident of low quality in this study, but of the high risk of bias concerning the results reported regarding the OPB-SM relationship.
Assessment of outcome	Use of shortened self-report questionnaire based on a validated measurement of OPB *****	Quote: "*The OPBS was shortened and modified for diary use. On a 5-point Likert-type scale ranging from 1 (Not at all) to 5 (Very much)"*

Criteria	**Assesment**	**With reference to:**
		Comment: the use of OPBS or a shortened version of it, is a strength. As to my knowledge, the OPBS is the only validated instrument for measuring OPB.
Statistical tests	Statistical test used to analyse the data were appropriate, clearly described and measures of association presented included confidence intervals and probability level (p value). *	Comment: descriptive data were reported, alongside confidence intervals and p-value. Adequate measures were conducted to answer the researchers` hypothesizes.
	**Erreygers, Vandebosch (64)**	
Representativeness of the sample	Truly representative of the average in the target population *	Quote: "*Participants were recruited through their schools. Schools were randomly selected from a province in Flanders. Twenty-nine schools were contacted, 13 of which agreed to participate … 1720 Dutch-speaking adolescents participated*".
Sample size	Not justified with sample size calculations, though satisfactory. *	Quote: see above.<br>Comment: no reported sample size calculation. However, the sample size is more than big enough to satisfy a conservative assumption about the nature of the true population value.
Non-respondents	No summary data on non-respondents, although it did handle missing data with statistical procedures. (*-)	Quote: *Maximum likelihood estimation was used to handle missing data.*<br>Comment: the authors only reported the method of handling missing data and not a summary of the missing data itself.
Ascertainment of the exposure	Adapted version of validated self-report measure on internet use.	Quote: "*Our survey contained a scale on Internet use based on items used in the Belgian version of the EU Kids Online (2014) questionnaire. On a 6-point scale, participants had to indicate how often they had used digital media in the past six months for 11 activities".*
Comparability	Not controlled for confounders/level of adjustment	Comment: the main aim of the study was not to investigate the SM-OPB relationship, and the variable of SM use in itself was included due to it being a possible confounder. Therefore, not adjusting for possible confounders in the association between SM and OPB is not evident of low quality in this study, but of the high risk of bias concerning the results reported regarding the OPB-SM relationship.
Assesment of outcome	Use of validated self-report measure of OPB *	*"We developed a scale to measure engagement in prosocial and antisocial behavior online. The scale consisted of two parts: The first part assessed which behaviors the adolescents had done themselves ("performing"), the second (equivalent) part assessed which behaviors the adolescents*

Criteria	**Assesment**	**With reference to:**
		had received from others (“receiving”). Each part consisted of 11 antisocial and 14 prosocial behaviors… The online prosocial behavior items consisted of five items adapted from the items used by Wright and Li (2011)… 9 items from two measures of offline prosocial behavior: Caprara and Pastorelli’s (1993) Prosocial Behaviour Scale and Carlo and Randall’s (2002) Prosocial Tendencies Measure… two items were poorly understood and we did not include them”. Comment: at that point, no validated instrument to measure OPB existed. The authors measured both giving and receiving OPB. However, no peer- or parental report of OPB, only self-report.
Statistical tests	Statistical test used to analyse the data were appropriate, clearly described and measures of association presented included confidence intervals and probability level (p value). *	Comment: descriptive data alongside confidence intervals and p-value, factor-analysis, structural equation model for testing association, and post-hoc for mediating variables, were described and properly conducted. The arguments for reporting and conducting the statistical tests were clear.

Note. This is a more detailed account of the process in which the risk of bias-assessment was based on. OPB = Online prosocial behavior, SM = Social media (use), OPBS = The Online Prosocial Behavior Scale.
